# Delusion of pregnancy : The role of prolactin

**DOI:** 10.1192/j.eurpsy.2022.1818

**Published:** 2022-09-01

**Authors:** R. Jomli, H. Jemli, H. Ghabi, Y. Zgueb, U. Ouali, A. Aissa

**Affiliations:** 1 Razi Hospital, Psychiatry A, manouba, Tunisia; 2 university of tunis elmanar, Faculty Of Medicine Of Tunis, manouba, Tunisia

**Keywords:** schizophrénia, delusion, Pregnancy, prolactine

## Abstract

**Introduction:**

A delusion of pregnancy is defined as a fixed false belief that one is pregnant in the absence of any evidence to support the pregnant state. Different mechanisms have been advanced to explain this phenomenon.

**Objectives:**

Herein, we present a case of hyperprolactinemia with delusion of pregnancy during the use of Amisulpride.

**Methods:**

Herein, we present a case of hyperprolactinemia with delusion of pregnancy during the use of Amisulpride.

**Results:**

This case concerns a 39-year-old, divorced Tunisian woman with the diagnosis of schizoaffective disorder,She has never been pregnant. She was taking Amisulpride at the dose of 400 mg per day with 100mg of Haldol Decanoate. In June 2021, she was admitted to our department as she expressed the thought that she was pregnant. No other psychotic symptoms were reported. There was no history of alcohol or illicit drug use. The physical examination was normal. The blood pregnancy test was negative. A measure of serum prolactin was performed and revealed a high prolactin level at 700 ng/ml. Amisulpride was discontinued. We then recorded the serum prolactin level at one month and then at three months after we stopped Amisulpride. We found that as her prolactin levels decreased her complaint of pregnancy also diminished. Actually, the patient has a normal level of prolactin and has no pregnancy delusion.

**Conclusions:**

Clinicians should be aware that delusion of pregnancy in psychotic patients may be caused by side effects of the treatment. Monitoring of serum prolactin levels in patients under Amisulpride may help to improve the management of these patients.

**Disclosure:**

No significant relationships.

